# Supplementary material for: Sex- and Age-Associated Differences in Genomic Alterations among Patients with Advanced Non-Small Cell Lung Cancer (NSCLC)
Source: Cancers (Basel). 2024 Jun 27;16(13):2366. doi: 10.3390/cancers16132366 (PMC11240325; doi:10.3390/cancers16132366)
Supplement: Supplementary file 1 [file cancers-16-02366-s001.zip › cancers-2983471-supplementary.pdf]

Supplementary Materials

Supplemental Table S1. Analysis of Co-Occurrence of Alterations of Interest Using cBioPortal.

Alteration A	Alteration B	Tendency	p-Value
ALK	ERBB2	Mutual exclusivity	<0.001
ALK	BRAF	Mutual exclusivity	0.002
ALK	PIK3CA	Mutual exclusivity	0.003
EGFR	KRAS	Mutual exclusivity	<0.001
EGFR	TP53	Mutual exclusivity	<0.001
EGFR	STK11	Mutual exclusivity	<0.001
EGFR	ALK	Mutual exclusivity	<0.001
EGFR	ERBB2	Mutual exclusivity	<0.001
EGFR	BRAF	Mutual exclusivity	<0.001
EGFR	MET	Mutual exclusivity	<0.001
EGFR	ROS1	Mutual exclusivity	<0.001
EGFR	PIK3CA	Mutual exclusivity	0.003
EGFR	RET	Mutual exclusivity	0.004
ERBB2	PIK3CA	Mutual exclusivity	<0.001
ERBB2	BRAF	Mutual exclusivity	0.003
KRAS	TP53	Mutual exclusivity	<0.001
**KRAS**	**STK11**	**Co-occurrence**	**<0.001**
KRAS	MET	Mutual exclusivity	<0.001
KRAS	ALK	Mutual exclusivity	<0.001
KRAS	ERBB2	Mutual exclusivity	<0.001
KRAS	BRAF	Mutual exclusivity	<0.001
KRAS	ROS1	Mutual exclusivity	<0.001
KRAS	PIK3CA	Mutual exclusivity	<0.001
KRAS	RET	Mutual exclusivity	0.019

MET	TP53	Mutual exclusivity	<0.001
MET	STK11	Mutual exclusivity	<0.001
MET	BRAF	Mutual exclusivity	<0.001
MET	PIK3CA	Mutual exclusivity	<0.001
MET	ERBB2	Mutual exclusivity	<0.001
MET	ALK	Mutual exclusivity	<0.001
STK11	ALK	Mutual exclusivity	<0.001
STK11	ERBB2	Mutual exclusivity	<0.001
STK11	BRAF	Mutual exclusivity	<0.001
STK11	PIK3CA	Mutual exclusivity	<0.001
STK11	ROS1	Mutual exclusivity	0.004
TP53	STK11	Mutual exclusivity	<0.001
TP53	ALK	Mutual exclusivity	<0.001
TP53	ERBB2	Mutual exclusivity	<0.001
TP53	BRAF	Mutual exclusivity	<0.001
TP53	PIK3CA	Mutual exclusivity	<0.001
TP53	ROS1	Mutual exclusivity	<0.001
TP53	RET	Mutual exclusivity	0.032

This table shows the results of the mutual exclusivity analysis performed using the publicly available cBioPortal (https://www.cbioportal.org/). We evaluated individuals with an alteration of interest (n=19,923). While most of these mutations tended to occur in isolation, *STK11* and *KRAS* were statistically likely to co-occur (p <0.001).

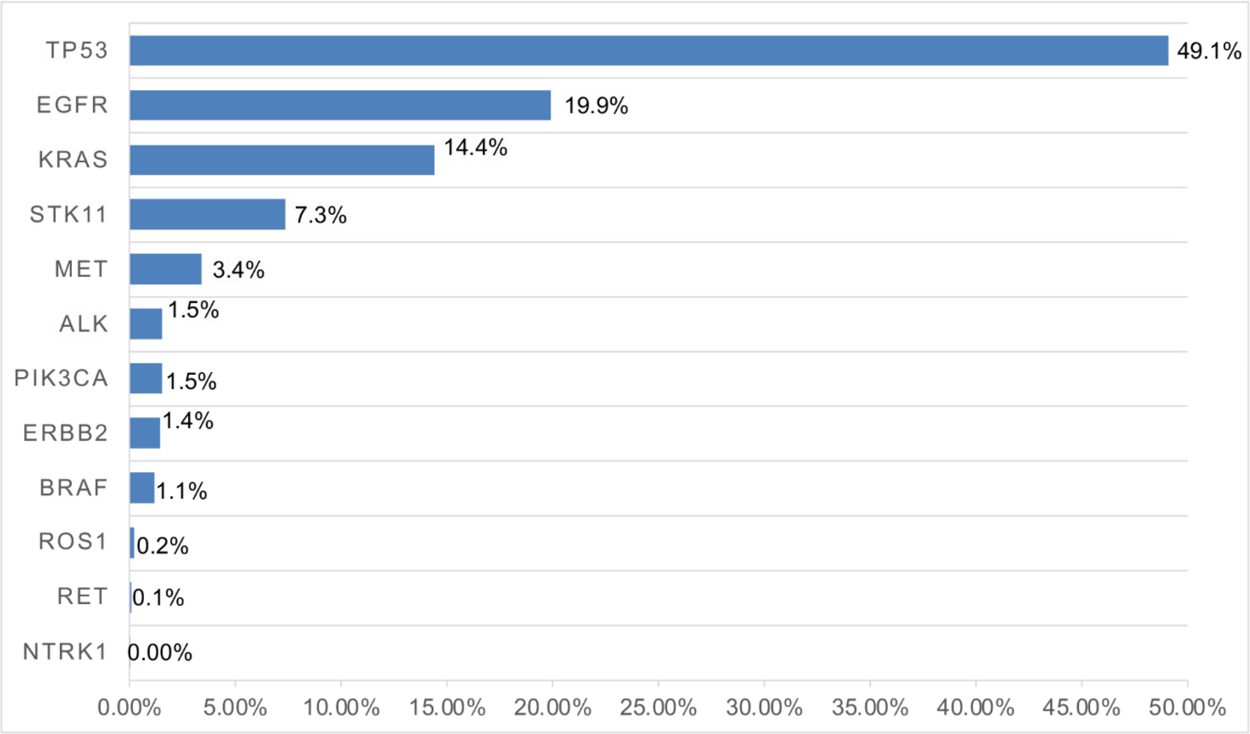

Supplemental Figure S1. Most Common Alterations of Interest.

Bar graph depicting the percentage of each alteration of interest detected (n = 19,923).

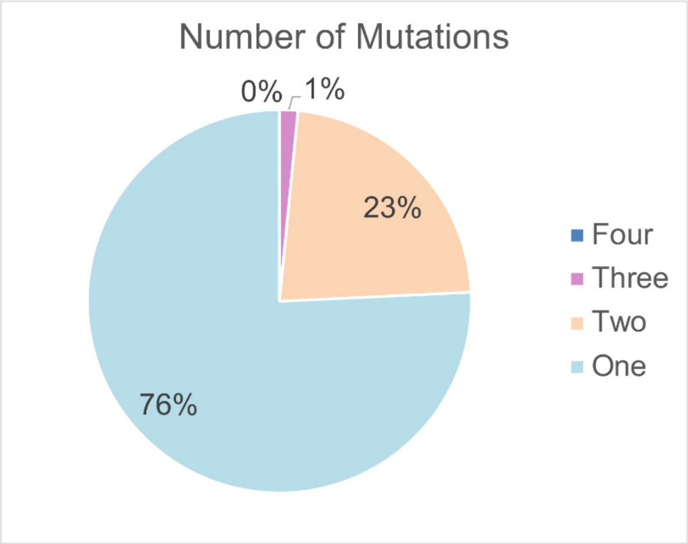

Four	6	0.03%
Three	304	1.53%
Two	4520	22.69%
One	15093	75.76%

Supplemental Figure S2. Number of Mutations Per Individual with an Alteration of Interest.

Pie chart demonstrating individuals with an alteration of interest (n = 19,923) who had one or more alterations (of interest).

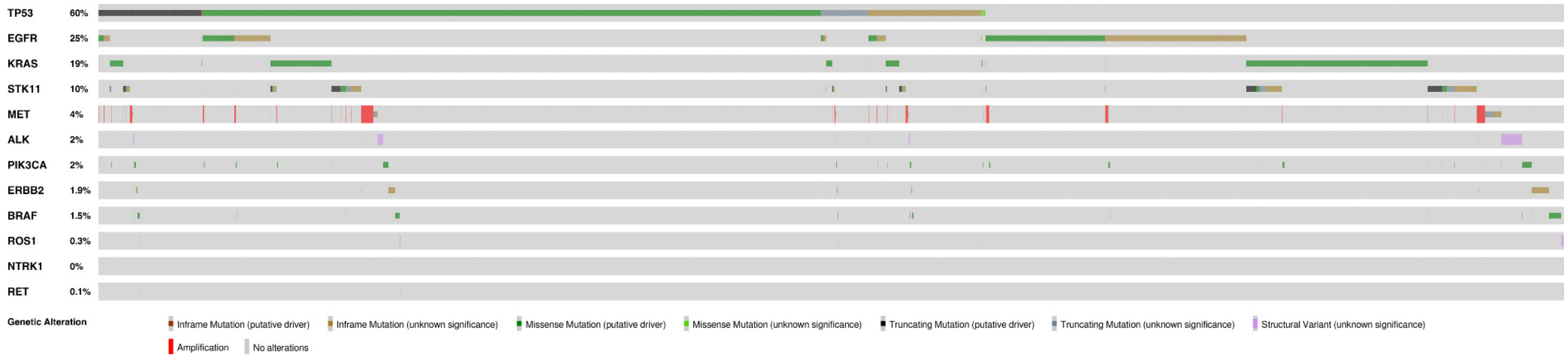

Supplemental Figure S3. Oncoprint.

This figure shows the co-occurring mutation profiles for patients with an alteration of interest (n = 19,923).
